# *TP53* wild-type*/PPM1D* mutant diffuse intrinsic pontine gliomas are sensitive to a MDM2 antagonist

**DOI:** 10.1186/s40478-021-01270-y

**Published:** 2021-11-03

**Authors:** Cheng Xu, Heng Liu, Christopher J. Pirozzi, Lee H. Chen, Paula K. Greer, Bill H. Diplas, Liwei Zhang, Matthew S. Waitkus, Yiping He, Hai Yan

**Affiliations:** 1 Department of Pathology, Duke University, Durham, NC USA; 2 Preston Robert Tisch Brain Tumor Center, Duke University Medical Center, 199B-MSRB Building, Research Drive, Durham, NC 27710 USA; 3 Department of Neurosurgery, Beijing Tiantan Hospital, Capital Medical University, Beijing, China; 4 Department of Radiation Oncology, Memorial Sloan Kettering Cancer Center, New York, NY USA; 5 Department of Neurosurgery, Duke University, Durham, NC USA

**Keywords:** Diffuse intrinsic pontine gliomas, RG7388, p53 pathway

## Abstract

Diffuse intrinsic pontine gliomas (DIPGs) are high-grade tumors of the brainstem that often occur in children, with a median overall survival of less than one year. Given the fact that DIPGs are resistant to chemotherapy and are not amenable to surgical resection, it is imperative to develop new therapeutic strategies for this deadly disease. The p53 pathway is dysregulated by *TP53* (~ 60%) or *PPM1D* gain-of-function mutations (~ 30%) in DIPG cases. PPM1D gain-of-function mutations suppress p53 activity and result in DIPG tumorigenesis. While MDM2 is a major negative regulator of p53, the efficacy of MDM2 inhibitor has not been tested in DIPG preclinical models. In this study, we performed a comprehensive validation of MDM2 inhibitor RG7388 in patient-derived DIPG cell lines established from both *TP53* wild-type/*PPM1D*-mutant and *TP53* mutant/*PPM1D* wild-type tumors, as well in *TP53* knockout isogenic DIPG cell line models. RG7388 selectively inhibited the proliferation of the *TP53* wild-type/*PPM1D* mutant DIPG cell lines in a dose- and time-dependent manner. The anti-proliferative effects were p53-dependent. RNA-Seq data showed that differential gene expression induced by RG7388 treatment was enriched in the p53 pathways. RG7388 reactivated the p53 pathway and induced apoptosis as well as G1 arrest. In vivo, RG7388 was able to reach the brainstem and exerted therapeutic efficacy in an orthotopic DIPG xenograft model. Hence, this study demonstrates the pre-clinical efficacy potential of RG7388 in the *TP53* wild-type/*PPM1D* mutant DIPG subgroup and may provide critical insight on the design of future clinical trials applying this drug in DIPG patients.

**Supplementary Information:**

The online version contains supplementary material available at 10.1186/s40478-021-01270-y.

## Introduction

Diffuse intrinsic pontine gliomas (DIPGs) are malignant brainstem gliomas. In the United States, there are approximately 200–300 new cases of DIPG every year [1]. DIPGs are resistant to conventional therapies, including radiation and chemotherapy, and are not amenable to surgical resection [2]. Consequently, DIPGs are universally lethal, with a median overall survival of less than one year [3, 4]. Novel therapies for DIPG are desperately needed.

The mutational landscape of DIPG has been well characterized. Recurrent somatic Lys-27-Met mutations in histone H3 genes (either in H3.1 or H3.3) have been identified as an oncogenic driver in ~ 80% of DIPGs [5–9]. In the latest 2016 World Health Organization classification, “diffuse midline glioma, H3K27M mutant” was introduced, and DIPGs are included in this new entity [10]. *TP53* and the genetic components of p53 signaling are also frequently mutated in DIPGs. *TP53* mutations were identified in around 60% H3K27M mutant DIPGs [11–15]. However, in *TP53* wild-type DIPGs, approximately 80% have mutations in exon 6 of *Protein Phosphatase, Mg2* + */Mn2* + *Dependent 1D* (*PPM1D*) mutations, which result in truncated PPM1D mutant proteins. The mutations stabilize the truncated PPM1D protein and lead to increases in its native dephosphorylation activity toward p53 and other proteins in DNA damage response (DDR) and cellular checkpoint pathways [11]. Recent studies in DIPGs have demonstrated that direct PPM1D inhibitor GSK2830371 inhibits p53 wild-type tumor cells growth and sensitizes cells to PARP inhibition and radiation through the re-activation of p53 pathway. These studies suggested that reactivation of p53 pathway is a feasible therapeutic strategy in DIPGs [16, 17].

Another approach to reactive p53 pathway is to reduce p53 protein ubiquitination and degradation by targeting Mouse double minute 2 homolog (MDM2), a key negative regulator of the p53 pathway. There are two major functions of MDM2: one as an E3 ubiquitin ligase of p53 and the other as an inhibitor of p53 transcriptional activation [18]. In addition, PPM1D dephosphorylates MDM2 at serine 395 and increases its affinity for p53. Dephosphorylated MDM2 protein inhibits p53 transcriptional activity and promotes p53 protein ubiquitination [19]. Several small molecule compounds have been recently developed targeting the MDM2-p53 axis to reactivate the tumor suppressor activity of p53. RG7388, also known as Idasanutlin, is a highly potent and selective second-generation MDM2 inhibitor which blocks the interaction between MDM2 and p53 [20]. Ongoing clinical trials using this orally bioavailable drug include a Phase III trial for acute myeloid leukemia (AML) and several Phase I/II trials in solid tumors [21, 22]. However, the preclinical and clinical evidence of using MDM2 inhibitors for treating DIPGs has not been well characterized. In this study, using a series of clinically and genetically relevant DIPG cell lines, we have investigated the therapeutic potential of using RG7388 for treating DIPGs.

## Materials and methods

### Analysis of cell lines CRISPR screening data

To identify the top dependent gene of *TP53* wild-type/*PPM1D*-mutant tumors, we integrate data from Mutations, Copy Number Alterations (Cancer Cell Line Encyclopedia, Broad, 2019) and CRISPR screening data of 808 cancer cell lines (Broad’s institute Cancer Dependency Map Public 21Q1 (https://depmap.org/portal/) [23]. These cell lines were classified into different molecular subgroups based on *TP53* and *PPM1D* status. We use Student’s t-test to compare the CERES score of each gene (18,116 genes total) in between molecular subgroups. These statistical analysis were performed in R, and 10% false-discovery rate (FDR, Benjamini-Hochberg) was applied using p.adjust function in R. Top dependency gene list were generated based on p value/adjusted *p* value and t value. CERES score of MDM2 across four subgroups were visualized with GraphPad Prism V9.0.

### Cell lines

Ten cell lines were obtained from biopsy or autopsy tissues of DIPG patient tumors. TT10714, TT10728 and TT10630 cell lines were used in a previous study [24]. SF7761 was a gift from Dr. C David James [25]. HSJD-DIPG-007, HSJD-DIPG-012 and HSJD-DIPG-013 were gifts from Dr. Angel Montero Carcaboso [26]. SU-DIPG-VI, SU-DIPG-XIII and SU-DIPG-35 were gifts from Dr. Michelle Monje [27]. Cell line authentication (CLA) analysis was performed at the Duke University DNA Analysis Facility and the result is shown in Additional file 6: Table S1. Primers for PCR amplification and sequencing of *H3F3A*, *TP53* and *PPM1D* genes are listed in Additional file 7: Table S2. HSJD-DIPG-007-NTC and HSJD-DIPG-*TP53* KO isogenic lines were established and used in a previous study [16].

### Western blot

Cell pellets were lysed and resolved using (4–12%) NuPAGE Bis–Tris gradient gel. Gels were soaked in NuPAGE protein transfer buffer and transferred to PVDF membranes. Membranes were blocked in room temperature for 1 h and incubated with primary antibodies at 4 degree overnight. Membranes were washed and then incubated with horseradish peroxidase (HRP) conjugated secondary antibody for one hour and HRP signals were detected by chemiluminescence using the BioRad ChemiDoc MP system. H3K27M (Cell Signaling Cat#74,829, 1:1000), p53 (Santa Cruz Cat#sc-126, 1:200), MDM2 (Cell Signaling Cat#86,934, 1:1000), p21 (Cell Signaling Cat#2947, 1:1000) and GAPDH (Santa Cruz Cat#sc-47724, 1:1000).

### Cell viability assay

Cells were plated at a density of 3000 cells/well in 96-well white and clear bottom microplates (Greiner Bio-One) and were incubated for 24 h. For dose-dependent studies, cells were treated with RG7388 (Selleck Chemicals #S7205) diluted in DMSO, ranging from 7.62 nM to 50 uM, or DMSO alone as a control, for 3 days. For time-dependent studies, cells were treated with DMSO, 50 nM and 100 nM RG7388 and luminescence signal was measured at Days 0, 2 and 4. Cell viability was measured by CellTiter-Glo Luminescent Cell Viability Assay (Promega #G7571) per manufacturer’s instruction. The luminescence signals were recorded using a Tecan Infinite M200 PRO microplate reader. Relative viabilities were calculated by normalizing luminescence values for each treatment condition to DMSO treated wells.

### RNA-seq

HSJD-DIPG-007-NTC and HSJD-DIPG-*TP53* KO were treated with 100 nM RG7388 or DMSO for 24 h. Three samples for each condition were harvested and RNA extractions were done using Maxwell RSC simplyRNA Cells Kit (Promega #AS1390). RNA samples were sent to for RNA-Seq and the resulting data was analyzed using the Galaxy/Europe platform [28].

### Cell apoptosis analysis

Cells were plated at a density of 3000 cells/well in 96-well white and clear-bottom microplates (Greiner Bio-One). After 24-h incubation, cells were treated with 100 nM RG7388 or DMSO for 24 h. Cell viability was measured by Caspase-Glo® 3/7 Assay (Promega #G8091) per manufacturer’s instruction. The luminescence signals were recorded using a Tecan Infinite M200 PRO microplate reader. Relative viabilities were calculated by normalizing luminescence values for each treatment condition to DMSO treated wells.

### Cell cycle analysis

HSJD-DIPG-007-NTC and HSJD-DIPG-*TP53* KO were treated with DMSO and 100 nM RG7388 for 24 h. 1 × 10^6^ Cells were harvested and fixed in cold 70% ethanol for 30 min at 4℃. After centrifugation and washing with PBS, 0.5 ml FxCycle PI/RNAse Staining Solution (ThermoFisher #F10797) was added to the cell pellet and incubated for 15–30 min at room temperature in the dark. Flow cytometry analysis was performed using 532-nm excitation with a 585/42-nm bandpass filter. Data was analyzed using FlowJo software.

### In vivo efficacy on mouse models

To access the pharmacokinetic of RG7388, 12 outbred athymic nude mice (J:NU Stock#007850) were treated with RG7388 delivered by oral gavage at 50 mg/kg. Plasma and tissues were collected and analyzed by Mass Spectrometry (AB Sciex 5500). To further access the in vivo efficacy of RG7388, 5 × 10^5^ of HSJD-DIPG-007 cells with CMV-Firefly luciferase lentivirus (Cellomics Technology cat#C839R47) were implanted stereotactically into the nude mouse brain stem region (n = 18). On the 21st-day post injection, mice were randomly assigned to two groups. For each group, nine mice were treated by oral gavage with 50 mg/kg of RG7388 (AstaTech cat#40916) or vehicle once per day, five days per week for three weeks. Mice were assessed for neurological symptoms and weight loss and sacrificed when either 20% weight loss was measured or when they exhibited neurologic symptoms. Kaplan–Meier curves were used for survival analysis.

### H&E and immunofluorescence staining

Six mice were injected with DIPG cells and treated with RG7388 (n = 3) or vehicle (n = 3) beginning on day 21 as previously described. These mice were sacrificed on day 42 and tumor samples were collected for immunofluorescence staining. The mouse brain was fixed in 10% formalin, embedded, and cut into sections. Standard H&E staining procedures and immunofluorescent staining procedures were performed. Immunofluorescent images were imaged on the Zeiss 880 at Duke Light Microscopy Core Facility. Positively stained cells were both manually counted and counted with ImageJ. Those assigned to counting were blinded to animal genotype and were given designated quadrants of specific size and magnification to count.

### More method details are in Supplementary Methods

## Results

### RG7388 selectively suppresses proliferation of *TP53* wild-type DIPG cells

**Fig. 1:**
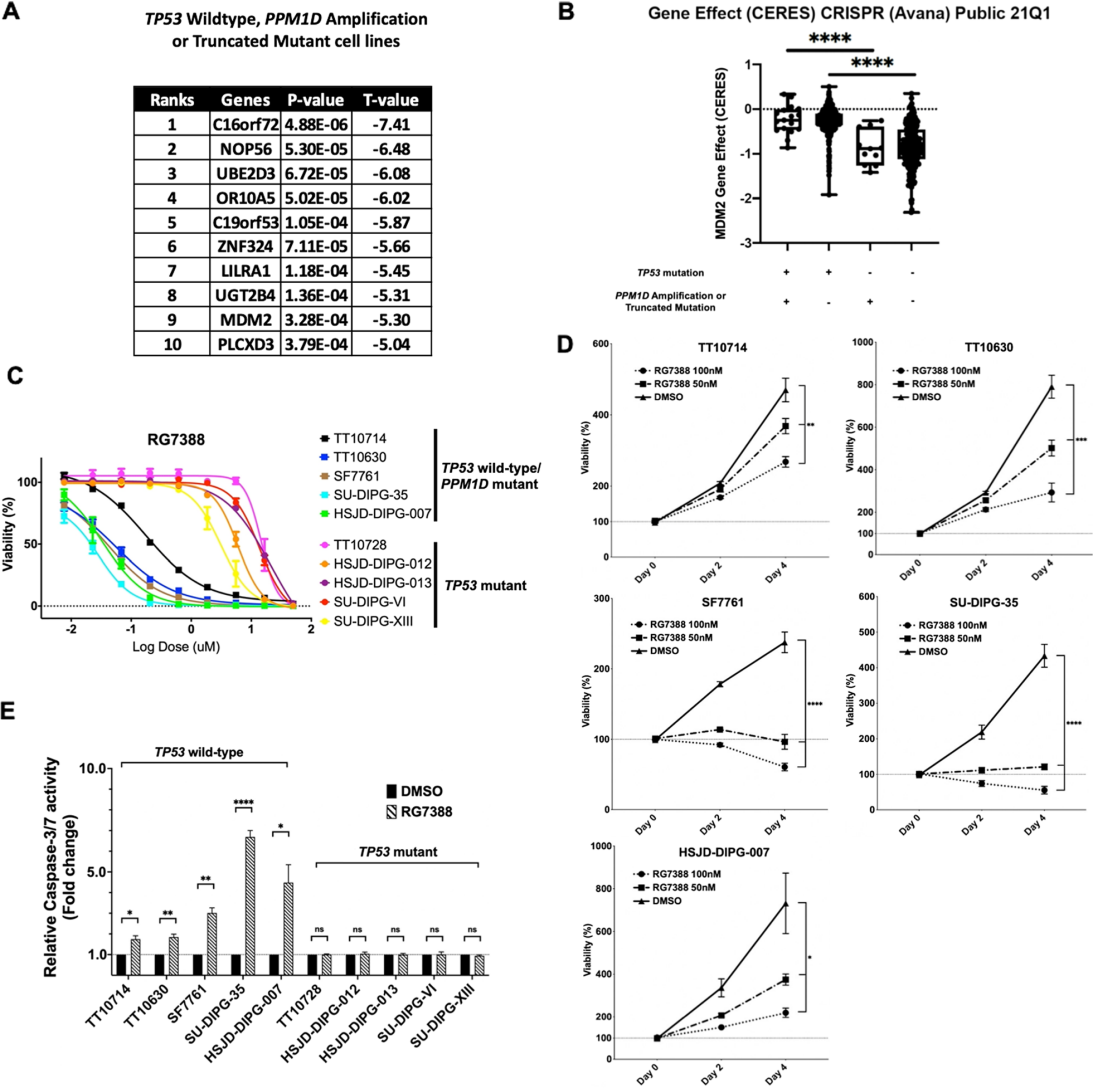
RG7388 selectively suppresses proliferation of TP53 wild-type DIPG cells. **A** Top dependent genes of *TP53* wildtype, *PPM1D* amplification or truncated mutant cell lines. **B** MDM2 Gene Effect (CERES) across four subgroups. **C** Ten DIPG cell lines were treated for three days with RG7388 at varying concentrations. Relative viabilities were calculated by normalizing luminescence values for each treatment condition to DMSO treated wells. **D** The viabilities of the five *TP53* wild-type DIPG lines treated with DMSO, 50 nM and 100 nM RG7388 for 2 and 4 days. Relative viabilities were calculated by normalizing luminescence values for each time point to Day 0 at the same treatment conditions. Mean ± SEM, *n* = 3 independent studies for each condition. *P* values based on Two-way ANOVA. *P < 0.05. **P < 0.01. ***P < 0.001. ****P < 0.0001. ns = non-significant. **E** Relative Caspase-3/7 activity after treatment with RG7388 for 24 h, normalized to DMSO treatment in *TP53* wild-type DIPG cell lines. Mean ± SEM, *n* = 3 independent studies for each condition. *P* values based on Student’s *t*-test. *P < 0.05. **P < 0.01. ****P < 0.0001. ns = non-significant

To validate MDM2 as a potential therapeutic target for *TP53* wild-type/*PPM1D* mutant cell lines, we combined data from Mutations, Copy Number Alterations (Cancer Cell Line Encyclopedia, Broad, 2019) and CRISPR screening data of 808 cancer cell lines (https://depmap.org/portal/; https://depmap.org/portal/; https://depmap.org/portal/) [23]. We determined that 543 cell lines harbor *TP53* mutation while 28 cell lines contain activated *PPM1D* either through *PPM1D* amplification or exon 6 truncations. Notably, among these 28 cell lines, 11 cell lines are *TP53* wild-type. To identify the top candidate genes for survival dependence in *TP53* wild-type cell lines, Student’s t-test was performed to compare gene effect (CERES score) across 18,116 genes in-between different molecular subgroups. Compared to all *TP53* mutant cell lines, *MDM2* is among the top ten dependent genes in *TP53* wild-type, *PPM1D* amplified or mutant cell lines (Fig. 1A). The *MDM2* gene effect CERES score is significant lower in *TP53* wild-type cell lines with/without *PPM1D* amplification mutation (Fig. 1B).

To further investigate the potential of targeting MDM2 in vitro, we tested RG7388, a second-generation inhibitor of MDM2 in ten DIPG patient-derived cell lines that were characterized genetic mutations common to DIPGs, including H3K27M, *TP53*, and *PPM1D* mutations (Additional file 1: Fig. S1). These experiments revealed that the *TP53* wild-type cell lines were significantly more sensitive to RG7388 (IC50 of 67 nM; range: 28–177 nM) compared to the *TP53* mutant cell lines (IC50 of 11.7 µM; range: 3.4–21.1 µM) (Student’s *t*-test, *P* < 0.0001, Fig. 1C, Additional file 2: Fig. S2A). Moreover, measured at two time points following treatment, both doses of RG7388 (50 nM and 100 nM) selectively suppressed the proliferation of the *TP53* wild-type cell lines, as compared to DMSO control (Fig. 1D). In contrast, the *TP53* mutant cell lines were resistant to both doses of RG7388, with no difference in cell numbers between treatment and DMSO control (Additional file 2: Fig. S2B). Treatments with RG7388 led to cell apoptosis in *TP53* wild-type DIPG cell lines but not in those without functional *TP53*, as indicated by the RG7388-induced activation of caspase-3/7 activities in the former but not the latter group of cell lines (Student’s *t*-test, p < 0.05, Fig. 1E). Collectively, these results established MDM2 as a top candidate for therapeutic targeting in *TP53* wild-type/*PPM1D* mutant cell lines, and suggested a strategy of leveraging MDM2 inhibitors to target the subset of DIPG with functional *TP53*.

### The susceptibility of DIPG cells to RG7388 is *TP53*-dependent

**Fig. 2:**
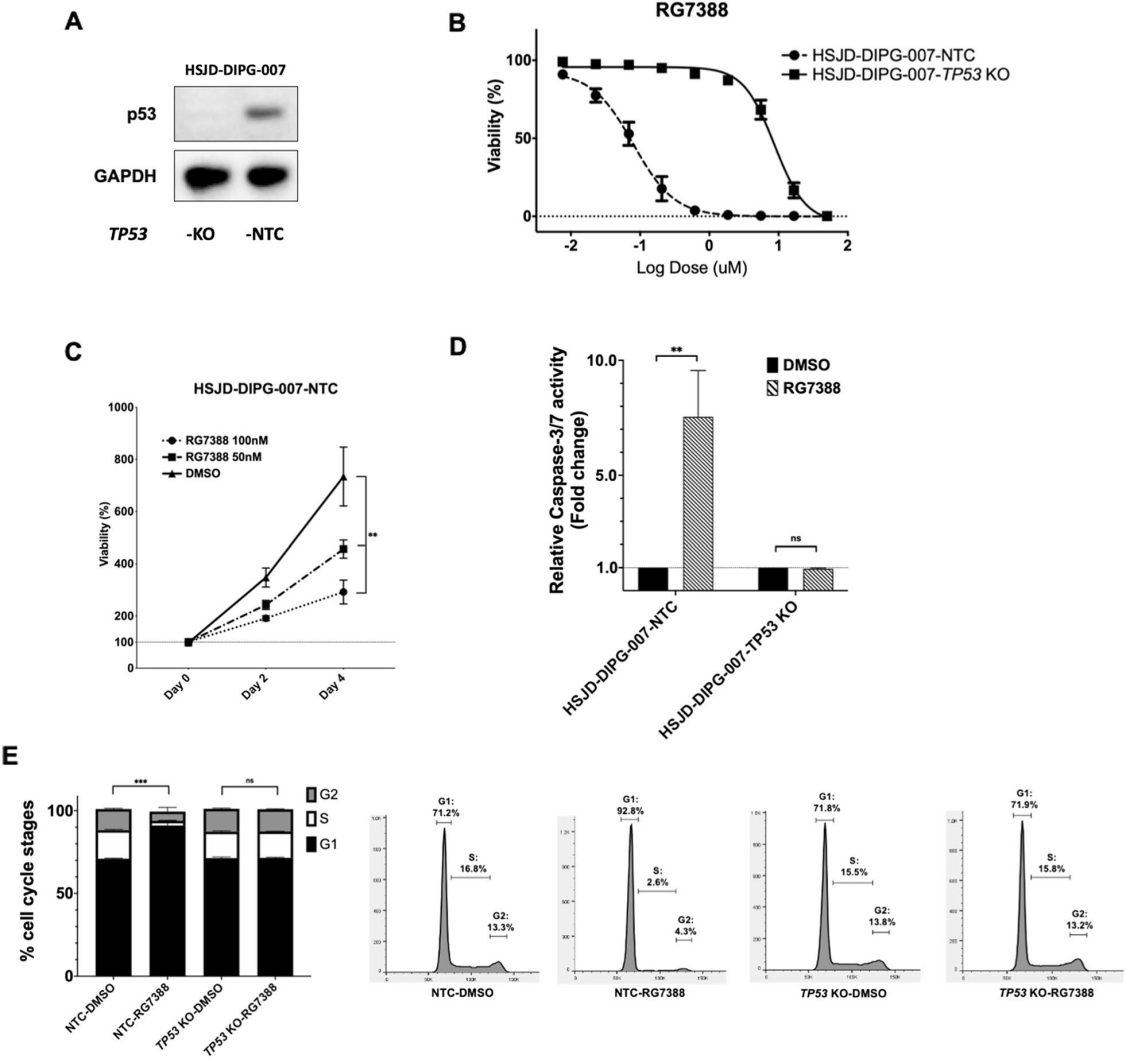
The susceptibility of DIPG cells to RG7388 is *TP53*-dependent. **A** The p53 expression of HSJD-DIPG-007-NTC (*TP53* wild-type) and HSJD-DIPG-007-*TP53* KO lines by western blot. **B** Cell viability of HSJD-DIPG-007-NTC and HSJD-DIPG-007-*TP53* KO lines measured after three days of RG7388 treatment. Relative viabilities were calculated by normalizing luminescence values for each treatment condition to DMSO treated wells. **C** The viabilities of HSJD-DIPG-007-NTC line treated with DMSO, 50 nM and 100 nM RG7388 for two and four days. Relative viabilities were calculated by normalizing luminescence values for each time point to Day 0 at the same drug concentration. Mean ± SEM, *n* = 3 independent studies for each condition. *P*-values based on Two-way ANOVA. **P < 0.01. **D** Relative Caspase-3/7 activity after treatment with RG7388 for 24 h, normalized to DMSO treatment in HSJD-DIPG-007-NTC and HSJD-DIPG-007-*TP53* KO lines. Mean ± SEM, *n* = 3 independent studies for each condition. *P*-values based on Student’s *t*-test. **P < 0.01. ns = non-significant. **E** Percentage of G1, S and G2 phases and representative images of HSJD-DIPG-007-NTC and HSJD-DIPG-007-*TP53* KO treated with DMSO or RG7388 as measured by PI staining. Mean ± SEM, *n* = 3 independent studies for each condition. *P* values based on chi-squared test. ***P < 0.001. ns = non-significant

To determine the roles of functional *TP53* in mediating the suppression effects of RG7388 on DIPG cells, we used an isogenic pair of HSJD-DIPG-007 cell line derivative with or without functional *TP53* [16], namely, a non-targeting control (NTC) sgRNA and *TP53* KO, respectively (Fig. 2A) and tested their susceptibility to RG7388. These experiments revealed that *TP53* KO conferred resistance to RG7388, as evidenced by the > 100 × IC50 for the *TP53* KO line when compared to the IC50 for the NTC line (8.931 µM vs. 86 nM, Student’s *t*-test, *p* < 0.01, Fig. 2B, Additional file 2: Fig. S2C). Consistent with these findings, RG7388 exerted both dose and time-dependent suppression effects on the control cell lines, but displayed no effects on the *TP5*3 KO cell line (Fig. 2C, Additional file 2: Fig. S2D). In further supporting the essential role of functional *TP53* in conferring the susceptibility to RG7388, HSJD-DIPG-007-NTC cells, but not the HSJD-DIPG-007-*TP53* KO counterparts, underwent both apoptosis (Fig. 2D) and G1 arrest (Fig. 2E) in response to RG7388 treatment.

**Fig. 3:**
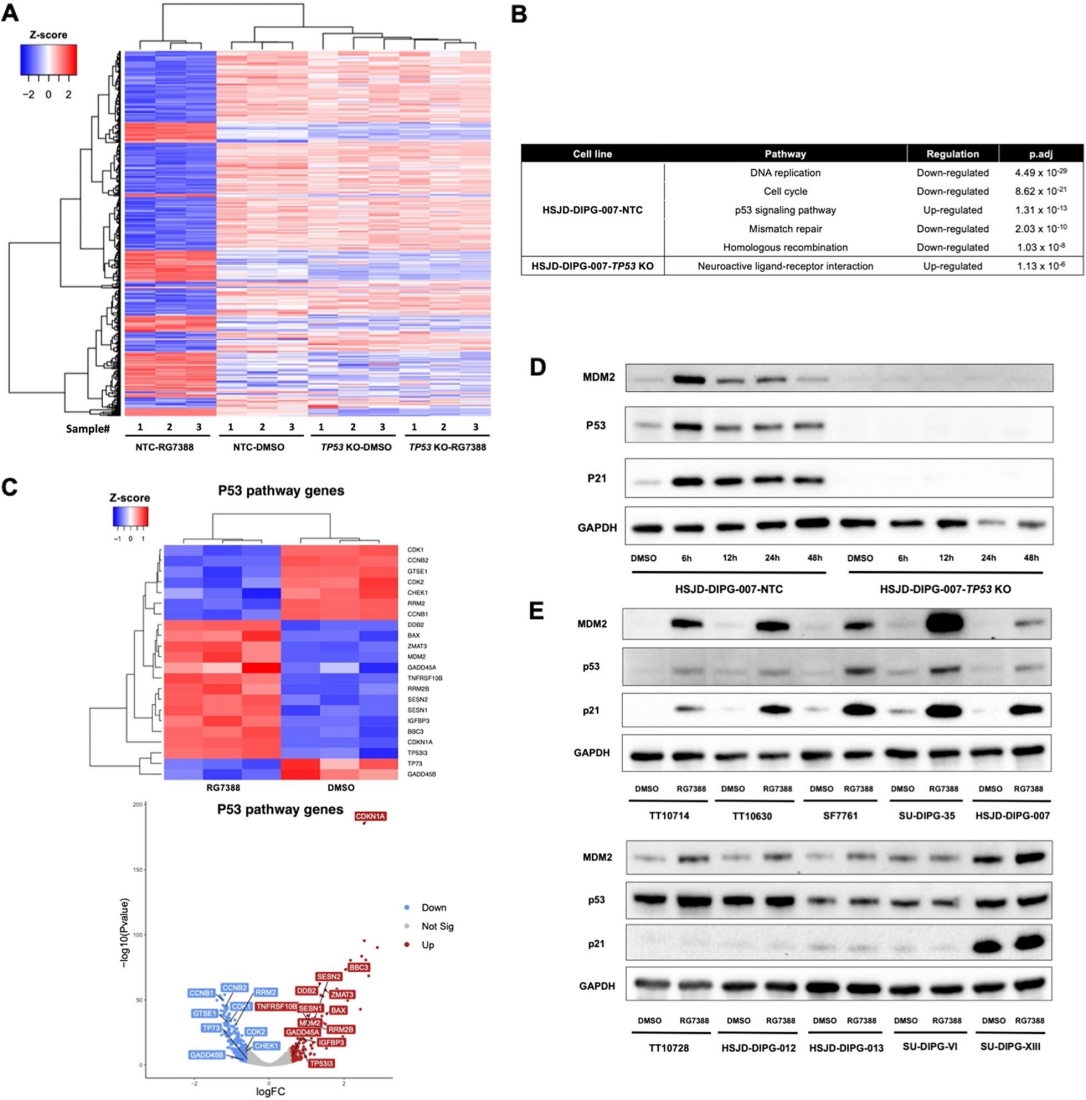
P53 pathway was up-regulated after RG7388 treatment. **A** Hierarchical clustering of significantly changed genes (adjusted *P*-values < 0.05, fold change > 1.5) of HSJD-DIPG-007-NTC and HSJD-DIPG-007-*TP53* KO lines after treatment with DMSO and RG7388. Columns in the heat map represent individual replicates (three for each condition). Rows represent genes, colored by log-transformed transcript intensity (DESeq2-normalized counts + 1) in z-score. Blue shows replicates with low expression (z-score < 0); red shows replicates with high expression (z-score > 0). **B** Top five most differentially regulated KEGG pathways of HSJD-DIPG-007-NTC and HSJD-DIPG-007-*TP53* KO lines response to RG7388. **C** Hierarchical clustering of significantly changed genes (adjusted *P*-values < 0.05, fold change > 1.5) within the p53 pathway of HSJD-DIPG-007-NTC response to RG7388. Columns in the heat map represent individual replicates (three for each condition). Rows represent genes, colored by log-transformed transcript intensity (DESeq2-normalized counts + 1) in z-score. Blue shows replicates with low expression (z-score < 0); red shows replicates with high expression (z-score > 0). Volcano plot of p53 pathway genes with significant change (adjusted *P*-values < 0.05, fold change > 1.5). Blue, red and gray show down-regulated, up-regulated and not significantly changed genes, respectively. **D** Protein expression level of MDM2, p53 and p21 in HSJD-DIPG-007-NTC and HSJD-DIPG-007-*TP53* KO treated with DMSO or 25 nM RG7388 for 6 h, 12 h, 24 h and 48 h. **E** Protein expression level of MDM2, p53 and p21 in five *TP53* wild-type (Top) and five *TP53* mutant (Bottom) DIPG cell lines treated with DMSO and 100 nM RG7388 for 24 h

To gain further insight into the mechanism underlying the effects of RG7388, we performed RNA-Seq analysis comparing the isogenic pair of derivatives, HSJD-DIPG-007-NTC and HSJD-DIPG-007-*TP53* KO, after treatment with DMSO or 100 nM RG7388 for 24 h. The differences in transcript level (FPKM) between RG7388 treatment and DMSO control were assessed, and differentially expressed genes (adjusted *P*-value < 0.05, fold change > 1.5) were selected. While the global gene expression was mostly unaffected in the HSJD-DIPG-007-*TP53* KO cell line, 476 genes were significantly changed in the HSJD-DIPG-007-NTC line (Fig. 3A). Gene sets enrichment analysis revealed the top five most significantly altered KEGG pathways in this cell line in response to RG7388 treatment (adjusted *P*-values ranging from 4.49 × 10^–29^ to 1.03 × 10^–8^) included the down-regulation of the pathways involving DNA replication, cell cycle, mismatch repair and homologous recombination and most notably, the upregulation of the p53 signaling pathway [29] (Fig. 3B). Hierarchical clustering analysis of genes in these pathways (adjusted *P*-value < 0.05, fold change > 1.5) (DESeq2-normalized counts + 1) revealed those related to cell cycle arrest and apoptosis were significantly changed, in that apoptosis related genes (e.g., *BAX*, *BBC3* (*PUMA*), *TNFRSF10B* (*DR5*) and *ZMAT3* (PAG608) were up-regulated while cell cycle progression related genes (e.g., *CDK1*, *CDK2*, *CCNB1* and *CCNB2*) were down-regulated (Fig. 3C, Additional file 3: Fig. S3A and S3B). Notably, *CDKN1A*, the gene encoding p21, exhibited the lowest adjusted *P*-value and displayed one of the highest fold-changes in response to treatment of genes in the p53 pathway (Additional file 3: Fig. S3C). To further corroborate the activation of the p53 pathway in the p53-intact cells, we treated HSJD-DIPG-007-NTC and *TP53* KO lines with RG7388 determined the expression of proteins in the p53 pathway at different time points post the treatment (Fig. 3D). These experiments revealed a rapid induction of the p53, p21 and MDM2 in response to RG7388, reaching peak levels at 6 h post the treatment, while the induction of these proteins was not detectable in the HSJD-DIPG-007-*TP53* KO after treatment with RG7388 (Additional file 4: Fig. S4A).

Finally, to further confirm the activation of the p53 pathway in response to RG7388 in DIPG cell lines with functional *TP53*, we treated the panel of the original DIPG cell lines with RG7388 for 24 h and determined the levels of MDM2, p53 and p21 proteins. These experiments confirmed that in the *TP53* mutant DIPG cell lines, the expression levels of MDM2, p53 and p21 displayed only minimal alterations, and in contrast, the levels of these proteins were all markedly increased compared to DMSO control in five *TP53* wild-type DIPG lines, with a mean fold change ranging from 19 to 63 (MDM2: Student’s *t*-test, *P* < 0.0001), 4 to 71 (p53: Student’s *t*-test, *P* < 0.0001) and 7 to 32 (p21: Student’s *t*-test, *P* < 0.0001) (Fig. 3E, Additional file 4: Fig. S4B).

Collectively, these results confirmed that RG7388 induced activation of the p53 pathway and that MDM2 inhibition-induced suppression of the DIPG cell lines was *TP53* dependent.

### RG7388 was able to reach the brainstem and exerted therapeutic efficacy in an orthotopic DIPG xenograft model

**Fig. 4:**
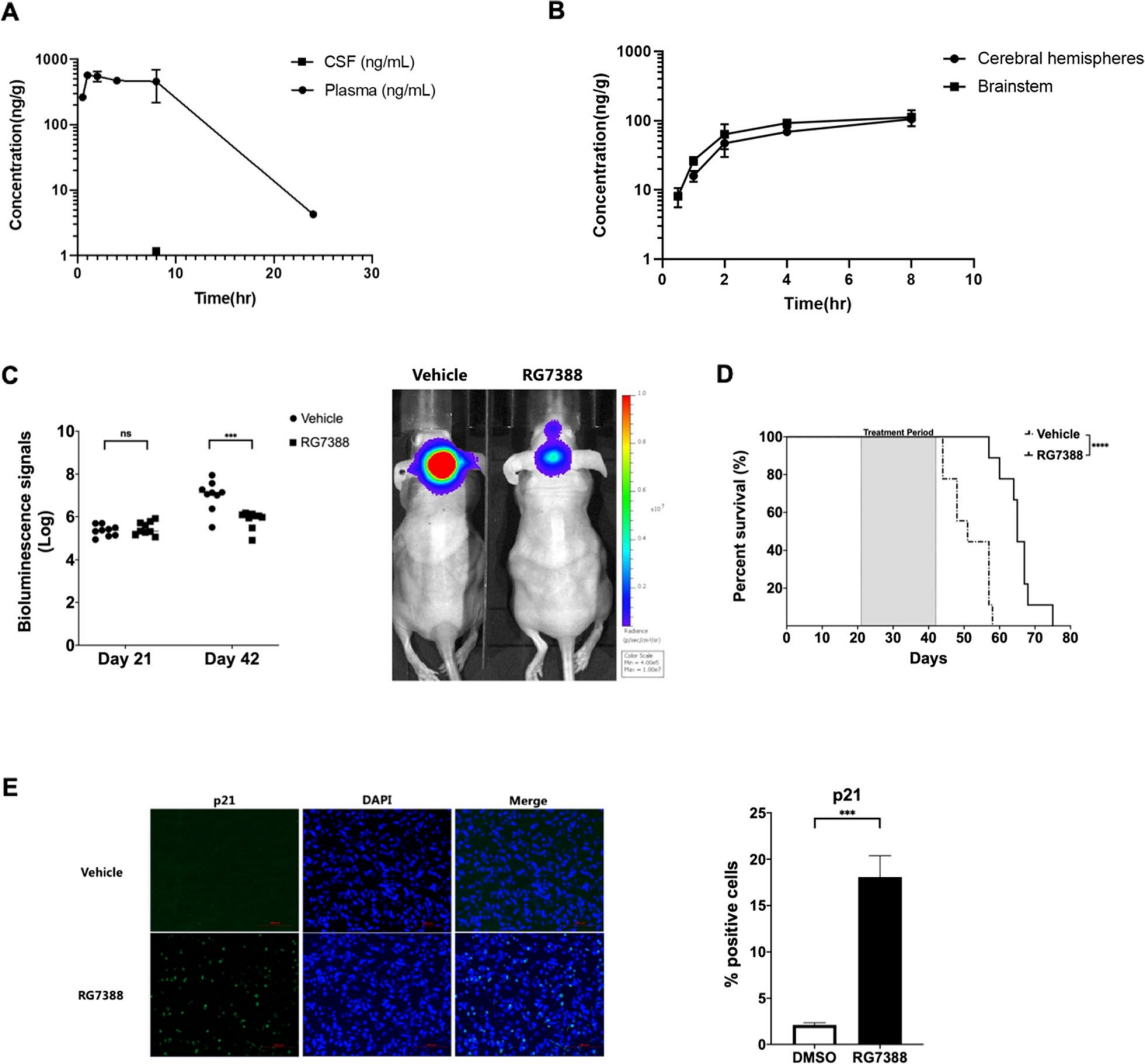
RG7388 was able to reach the brainstem and exert therapeutic efficacy in an orthotopic DIPG xenograft model. **A** The pharmacokinetic profile shows the RG7388 concentration level in plasma and CSF (mean ± SEM, n = 2 per time point). **B** The pharmacokinetic profile shows the RG7388 concentration level in cerebral hemispheres and brainstem (mean ± SEM, n = 2 per time point). **C** Log values of bioluminescence signals for each group on day 21 and day 42 respectively (left). Acquired on day 42, representative bioluminescence signal of two mice assigned to treatment and vehicle group (right). *P*-values based on Student’s t-test. ***P < 0.001. ns = non-significant. **D** Kaplan–Meier curve of treatment and vehicle group. N = 9 for each group. *P*-values based on log-rank test. ****P < 0.0001. **E** The expression of p21 of mouse brain samples from vehicle or RG7388 treatment group on day 42. Scale bar: 100 μm. Mean ± SEM, n = 3 for each group*. P*-values based on chi-squared test. ***P < 0.001

Permeability to the brainstem poses a major challenge for a drug’s application for treating DIPGs. To determine the applicability of RG7388 for treating DIPG, we determined the permeability of orally administered RG7388 into the brainstem in mice. We administered RG7388 via oral gavage (50 mg/kg) and collected blood samples, cerebrospinal fluid (CSF), cerebral hemispheres tissues and brainstem tissues from the mice at different time points (0.5/1/2/4/8/24 h) afterward and analyzed the presence and concentrations of RG7388 by mass spectrometry. The brains were perfused to remove blood before the submission for mass spectrometry. RG7388 was detectable in plasma with mean concentration of 263 ng/ml 30 min after the drug administration. Plasma concentration increased to the maximum (mean 570 ng/ml) after 1 h, remained relatively steady at least for the following 7 h, and decreased dramatically by 24 h (4.26 ng/ml) (Fig. 4A). Unlike in the plasma, RG7388 was mostly not detectable in the CSF, as it was detected at a low concentration (average 1.17 ng/ml) after 8 h on in mouse. Notably, RG7388 was present in the cerebral hemispheres and in the brainstem rapidly after its administration, measured at an average of ~ 18 ng and 8.1 ng per gram of tissues in the cerebral hemispheres and in the brainstem, respectively, within one hour post its administration. The accumulation of RG7388 subsequently increased to the maximum level of 105 ng and 112 ng per gram of tissues in the cerebral hemispheres and brainstem, respectively (Fig. 4B). The peak permeability of the drug to the brainstem reached at the 8-h time point, with a ratio of RG7388 concentration in the brainstem to plasma of 0.292. RG7388 was not detectable either in brainstem or cerebral hemispheres at the 24-h time point. Collectively, these results from the pharmacokinetics assays suggest RG7388 is permeable to the brainstem independent of tumor-mediated blood–brain–barrier disruption.

We then assessed the in vivo therapeutic efficacy of RG7388 in a DIPG orthotopic model derived from the HSJD-DIPG-007 cell line. Tumor cells expressing luciferase were injected into the brainstem of NSG mice, and the tumor development was monitored by the Luciferase imaging system. Host mice displaying similar bioluminescence signal on day 21 post-injection were randomly assigned to vehicle or RG7388 treatment groups. RG7388 or vehicle was orally administered once per day, five days per week for three weeks (Day 42 post-implantation) and the progression of tumors was determined. No overt toxicity to other organs was observed during the experiment. Notably, while the bioluminescence of orthotopic tumors was comparable between groups before the treatment (on Day 21), three weeks of RG7388 treatment resulted in measurably suppressed tumor progression, as demonstrated by reduced bioluminescence signals (Student’s *t*-test, p < 0.001) (Fig. 4C). Subsequently, mice were sacrificed when either 20% weight loss was measured or when they exhibited neurologic symptoms. Kaplan–Meier survival analysis found mice in the RG7388 treatment group had a significantly better survival than those in the vehicle group (Fig. 4D, median survival 65 days vs. 51 days, *p* < 0.0001).

To assess the P53 pathway status after drug treatment, three mice for each RG7388 and vehicle group were sacrificed on day 42 after injection, and their tumor tissues were analyzed by H&E staining and immunofluorescence. H&E and H3K27M immunofluorescence staining confirm the presence of tumor cells in the brainstem region (Additional file 4: Fig. S4C). Furthermore, the activation of the p53 pathway was confirmed in the RG7388-treated tumors, as evidenced by the increased expression of p21 in RG7388-treated tumors in comparison to the control tumors (Fig. 4E). Collectively, results from these in vivo experiments suggest that RG7388 treatment activates the p53 pathway in orthotopic DIPG and displays therapeutic efficacy by prolonging survival of animals relative to vehicle treated controls.

## Discussion

Given the key role of MDM2 in p53 regulation, targeting p53-MDM2 axis to stabilize and activate p53 has been explored as a novel therapeutic strategy for human cancers [30]. The pyrrolidine compound RG7388 is a second-generation MDM2 small molecular inhibitor, which blocks the physical interaction between MDM2 and p53 and is undergoing pre-clinical and clinical evaluation as a potential anti-cancer therapeutic [20]. However, due to the scarcity of pre-clinical DIPG models, especially the *TP53* wild-type subtype, the in vitro and in vivo efficacy of RG7388 had not been studied in DIPG models. Hence in this study, we performed a comprehensive validation of RG7388 in five *TP53* wild-type and five *TP53* mutant patient-derived DIPG cell lines, as well as the engineered *TP53* knockout isogenic lines. Nanomolar levels of RG7388 suppressed cell proliferation, altered gene expression patterns, and induced cell apoptosis and G1 arrest in the *TP53* wild-type DIPG cell lines, indicating its high potency. *TP53* mutant/knockout DIPG cell lines were resistant to RG7388 with approximately 100 times higher IC50 and did not show significant alterations in gene expression patterns, cell apoptosis or cell cycle variation in response to the inhibitor treatment, representing RG7388’s high selectivity. These results demonstrate the promising clinically therapeutic potential of RG7388 in treating *TP53* wild-type DIPG tumors.

Reactivation of the p53 pathway is a critical indicator for the effectiveness of MDM2 inhibitors and a potential pharmacodynamic biomarker for MDM2 inhibitor response. In this study, we evaluated the re-activation of p53 function in response to RG7388 by various perspectives. First, gene expression level analysis was performed on RNA-seq data, showing that the p53 pathway was one of the top three most differentially regulated pathways after RG7388 treatment in the *TP53*-NTC line. Notably, *CDKN1A*, which encodes the key p53 pathway downstream marker p21, was dramatically up-regulated after the drug treatment. Second, protein expression level of MDM2, p53 and p21 was assessed and indicated that all of these proteins were up-regulated in response to RG7388 in *TP53* wild-type lines. Interestingly, while the protein level of p53 was significantly increased, mRNA expression of *TP53* was unchanged after drug treatment, indicating that p53 protein expression is likely regulated through post-transcriptional mechanisms [31]. In contrast, MDM2 and p21 demonstrated significant up-regulation in both gene and protein expression levels upon MDM2-p53 interaction inhibition with RG7388. Third, induction of apoptosis, as one of the hallmarks of the re-activation of p53 pathway, was evaluated [32]. RNA-seq data demonstrated increased apoptosis-related genes such as *BAX*, *PUMA*, *DR5* and *ZMAT3* in the *TP53*-NTC cell line compared to the KO line. Similarly, all patient-derived *TP53* wild-type/*PPM1D* mutant DIPG cells lines displayed significantly high level of caspase-3/7 activity in response to RG7388 treatment, compared to their *TP53* mutant counterparts. Finally, cell cycle alteration, another characteristic phenotype of p53 re-activation, was assessed [32]. Cell cycle regulation was shown to be the second most differentially altered pathway by RNA-seq gene analysis and key G2/M markers such as *CDK1*, *CDK2*, *CCNB1* and *CCNB2* were all down-regulated after RG7388 treatment in the *TP53*-NTC line. In the same cell line, indications of G1 arrest by PI staining were observed, which was absent in the *TP53*-KO setting. In summary, these data confirm the efficacy of RG7388 in re-activating the p53 pathway in *TP53* wild-type DIPG cell lines.

One of major concerns for brain tumor drug treatment is the penetration of blood–brain barrier (BBB). A previous study has shown the effects of RG7388 treatment in glioblastoma orthotopic models [30]. However, the BBB in the brainstem area is generally regarded as more intact for DIPG as compared with the BBB in supratentorial tumors [33], thus the penetration of RG7388 to brainstem remained unclear. In this study, we confirmed that RG7388 was able to reach brainstem even without the tumor-mediated blood–brain–barrier disruption and maintained a relatively high level of concentration for at least 8 h. Moreover, we used DIPG orthotopic models to assess the in vivo efficacy of RG7388 and demonstrated its ability to inhibit tumor growth through a three-week drug administration period. As a result, treatment with RG7388 not only prolonged survival time, but also increased the expression of p21, the hallmark of p53 pathway activation.

Based on our data, RG7388 could be a valuable therapeutic approach to develop clinically in the treatment of *TP53* wild-type/*PPM1D*-mutant DIPG subgroup as a p53 pathway re-activation drug. One of the merits of RG7388 is that its safety and feasibility have already been confirmed by a variety of clinical trials, including a Phase III trial. RG7388 is currently undergoing clinical trials alone or in combination with other drugs for acute myeloid leukemia (phase I/II: NCT02670044, NCT03850535; phase III: NCT02545283), myeloma (phase I/II: NCT02633059), lymphoma (phase I/II: NCT03135262), breast cancer (phase I/II: NCT03566485), a combination of acute leukemias and solid tumors (phase I/II: NCT04029688) and glioblastoma bearing an unmethylated MGMT promoter (phase I/II: NCT03158389). In general, RG7388 is well-tolerated by patients and the most common adverse events are limited to gastrointestinal system symptoms (diarrhea and vomiting), infections and myelosuppression [21, 22].

Several potential limitations of this MDM2-targeting therapeutic strategy are noted. First, in *TP53* wild-type DIPGs, *the* mutant PPM1D can stabilize MDM2 and promotes MDM2-mediated p53 ubiquitination and degradation. Thus, inhibition of PPM1D may further potentiate the efficacy of RG7388. A small-molecule PPM1D inhibitor, GSK2830371, was recently shown to enhance DNA damage response and suppress cell growth in DIPGs [16]. Further studies testing the combination therapy of GSK2830371 and RG7388 are warranted. Second, we speculate that the pressure from the prolonged treatment with RG7388 may lead to the selection and expansion of p53 mutant cells and thus the development of secondary resistance [34]. Additional combination treatment approaches, including radiation therapy, chemotherapeutic agents [35], the CDK4 inhibitors palbociclib [36], the PARP inhibitors rucaparib [37] and the MEK inhibitor trametinib [30], may be needed to maximize the eradication of tumor cells and mitigate the tumor recurrence. Finally, instead of oral administration, other novel drug delivery techniques, such as nanoparticle application [38] and convection-enhanced delivery (CED) [39], may offer superior local drug deliveries to further improve the therapeutic efficacy of RG7388.

## Supplementary Information

**Additional file 1: Figure S1.** (A) The characterization of ten patient-derived DIPG cell lines. Five cell lines (on the top) harbored H3K27M mutation and *PPM1D* truncating mutations, but were *TP53* wild-type. The other five cell lines (on the bottom) harbored mutant H3K27M and *TP53*, but did not contain mutation in exon 6 of *PPM1D*. (B) Protein expression level of H3K27M and p53 by Western Blot in ten patient-derived DIPG cell lines. (C) Sanger sequencing result of H3K27M mutation. (D) Sanger sequencing result of *TP53* mutation in SU-DIPG-XIII line. (E) Sanger sequencing result of *PPM1D* mutation in five *TP53* wild-type DIPG cell lines.

**Additional file 2: Figure S2.** (A) IC50 of RG7388 in five *TP53* wild-type and five *TP53* mutant DIPG lines. (B) The viabilities of the five *TP53* mutant DIPG lines treated with DMSO, 50nM and 100nM RG7388 for two and four days. Relative viabilities were calculated by normalizing luminescence values for each time point to Day 0 at the same treatment conditions. Mean ± SEM, n=3 independent studies for each condition. P-values based on Two-way ANOVA. ns=non-significant. (C) IC50 of RG7388 in HSJD-DIPG-007 NTC and *TP53* KO lines. (D) The viabilities of HSJD-DIPG-007 *TP53* KO line treated with DMSO, 50nM and 100nM RG7388 for two and four days. Relative viabilities were calculated by normalizing luminescence values for each time point to Day 0 at the same treatment conditions. Mean ± SEM, *n*=3 independent studies for each condition.* P*-values based on Two-way ANOVA. ns=non-significant.

**Additional file 3: Figure S3.** (A) Top five most differentially regulated KEGG pathways of HSJD-DIPG-007-NTC and HSJD-DIPG-007-*TP53* KO lines response to RG7388. (B) Hierarchical clustering of significantly changed genes (adjusted* P*-values < 0.05, fold change > 1.5) within the DNA replication and cell cycle pathway of HSJD-DIPG-007-NTC response to RG7388. Columns in the heat map represent individual replicates (three for each condition). Rows represent genes, colored by log-transformed transcript intensity (DESeq2-normalized counts + 1) in z-score. Blue shows replicates with low expression (z-score < 0); red shows replicates with high expression (z-score > 0). (C) Transcriptional levels of MDM2, TP53 and CDKN1A after RG7388 treatment in HSJD-DIPG-007-NTC line.

**Additional file 4: Figure S4.** (A) Quantification of protein expression level of MDM2, p53 and p21 in HSJD-DIPG-007-NTC and HSJD-DIPG-007-*TP53* KO treated with DMSO or 25nM RG7388 for 6 hours, 12 hours, 24 hours and 48 hours. Mean ± SEM, n=3 independent studies for each condition.* P*-values based on Two-way ANOVA. *P < 0.05. ***P < 0.001. ****P < 0.0001. (B) Quantification of protein expression level of MDM2, p53 and p21 in five* TP53* wild-type and five* TP53* mutant DIPG cell lines treated with DMSO and 100nM RG7388 for 24 hours. Mean ± SEM,* n*=3 independent studies for each condition.* P*-values based on Student’s t-test. ****P < 0.0001. (C) Representative H&E and immunofluorescence staining of brainstem xenograft. Scale bar: 100μm.

**Additional file 5:** Supplementary Methods

**Additional file 6: Supplymentary Table S1.** CLA analysis of tem DIPG cell lines

**Additional file 7: Supplymentary Table S2.** Primers for Sanger sequencing

## Data Availability

The datasets used and analyzed during the current study available from the corresponding author on reasonable request.
