# Supplementary figures and images for: TP53 wild-type/PPM1D mutant diffuse intrinsic pontine gliomas are sensitive to a MDM2 antagonist
Source: Acta Neuropathol Commun. 2021 Nov 3;9:178. doi: 10.1186/s40478-021-01270-y (PMC8565061; doi:10.1186/s40478-021-01270-y)

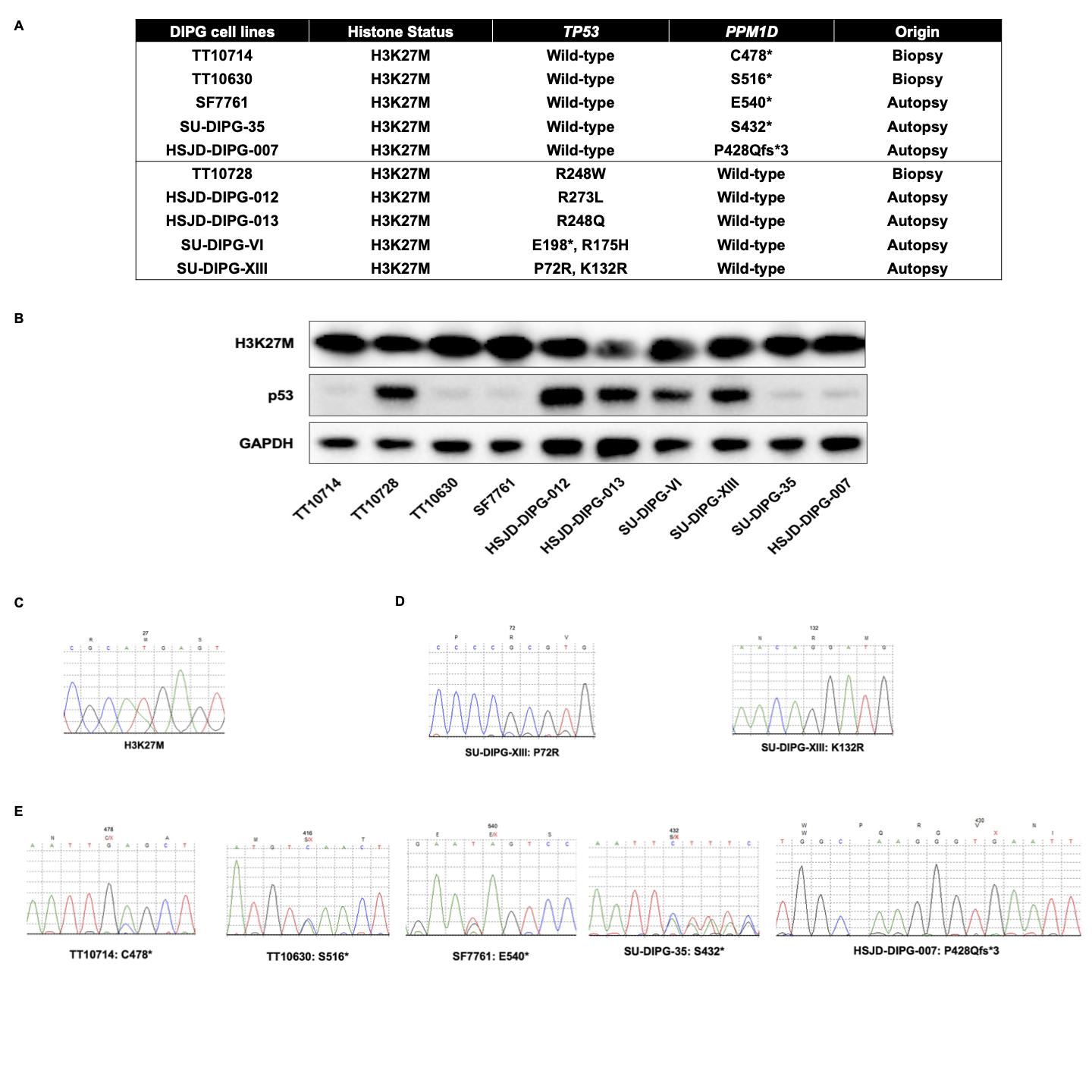

Supplement: Supplementary file 1 — Additional file 1: Figure S1. (A) The characterization of ten patient-derived DIPG cell lines. Five cell lines (on the top) harbored H3K27M mutation and PPM1D truncating mutations, but were TP53 wild-type. The other five cell lines (on the bottom) harbored mutant H3K27M and TP53, but did not contain mutation in exon 6 of PPM1D. (B) Protein expression level of H3K27M and p53 by Western Blot in ten patient-derived DIPG cell lines. (C) Sanger sequencing result of H3K27M mutation. (D) Sanger sequencing result of TP53 mutation in SU-DIPG-XIII line. (E) Sanger sequencing result of PPM1D mutation in five TP53 wild-type DIPG cell lines. [file 40478_2021_1270_MOESM1_ESM.tiff]

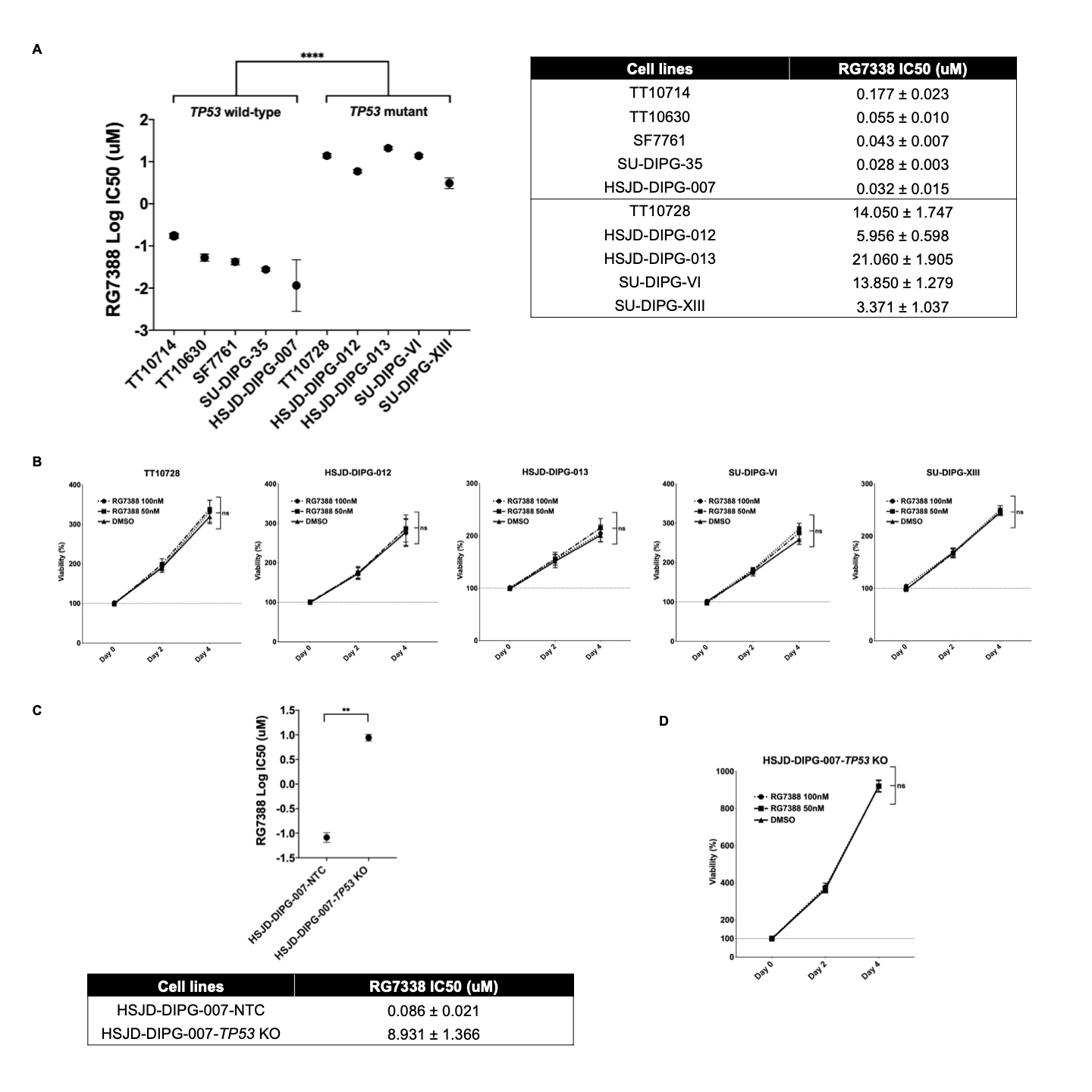

Supplement: Supplementary file 2 — Additional file 2: Figure S2. (A) IC50 of RG7388 in five TP53 wild-type and five TP53 mutant DIPG lines. (B) The viabilities of the five TP53 mutant DIPG lines treated with DMSO, 50nM and 100nM RG7388 for two and four days. Relative viabilities were calculated by normalizing luminescence values for each time point to Day 0 at the same treatment conditions. Mean ± SEM, n=3 independent studies for each condition. P-values based on Two-way ANOVA. ns=non-significant. (C) IC50 of RG7388 in HSJD-DIPG-007 NTC and TP53 KO lines. (D) The viabilities of HSJD-DIPG-007 TP53 KO line treated with DMSO, 50nM and 100nM RG7388 for two and four days. Relative viabilities were calculated by normalizing luminescence values for each time point to Day 0 at the same treatment conditions. Mean ± SEM, n=3 independent studies for each condition. P-values based on Two-way ANOVA. ns=non-significant. [file 40478_2021_1270_MOESM2_ESM.tiff]

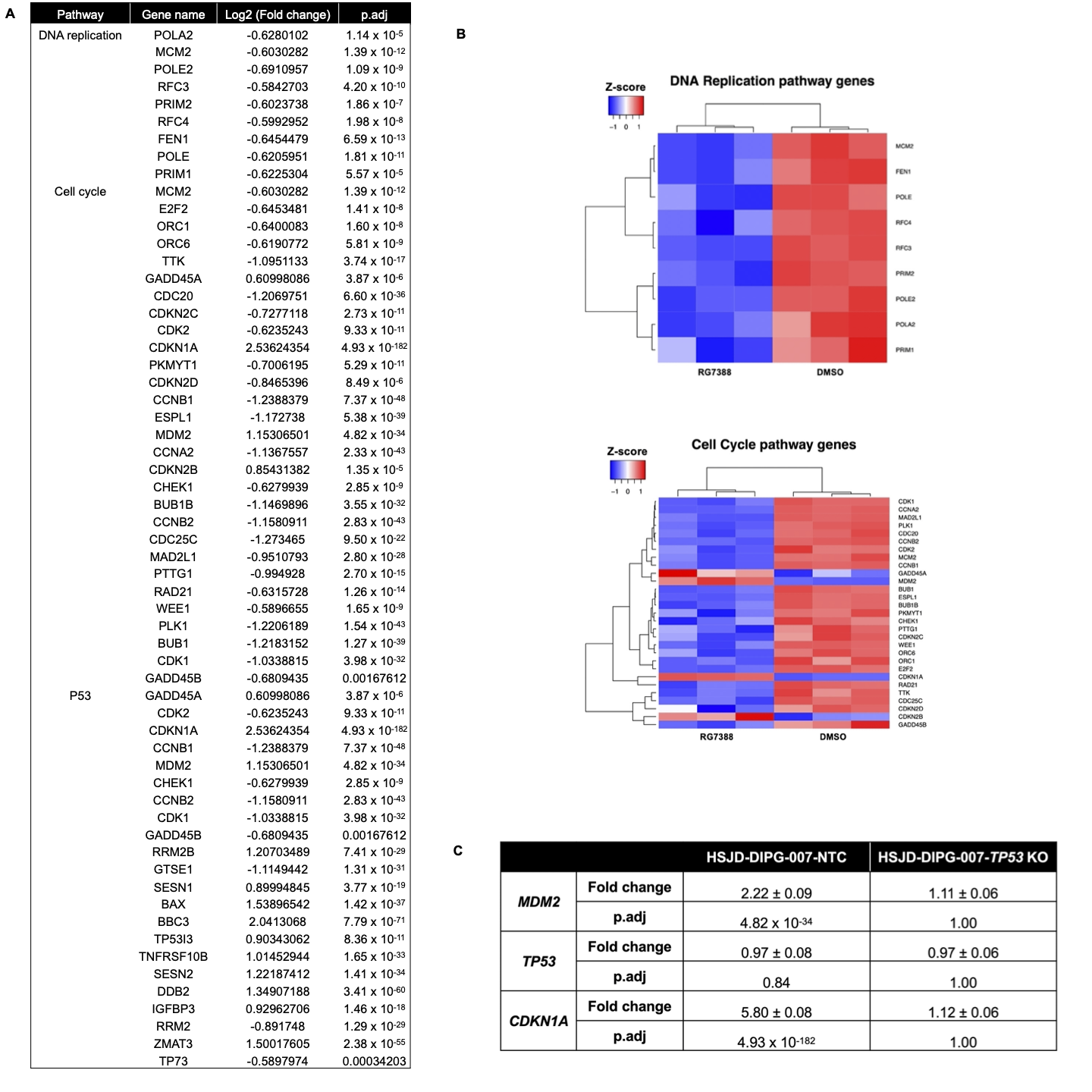

Supplement: Supplementary file 3 — Additional file 3: Figure S3. (A) Top five most differentially regulated KEGG pathways of HSJD-DIPG-007-NTC and HSJD-DIPG-007-TP53 KO lines response to RG7388. (B) Hierarchical clustering of significantly changed genes (adjusted P-values < 0.05, fold change > 1.5) within the DNA replication and cell cycle pathway of HSJD-DIPG-007-NTC response to RG7388. Columns in the heat map represent individual replicates (three for each condition). Rows represent genes, colored by log-transformed transcript intensity (DESeq2-normalized counts + 1) in z-score. Blue shows replicates with low expression (z-score < 0); red shows replicates with high expression (z-score > 0). (C) Transcriptional levels of MDM2, TP53 and CDKN1A after RG7388 treatment in HSJD-DIPG-007-NTC line. [file 40478_2021_1270_MOESM3_ESM.tiff]

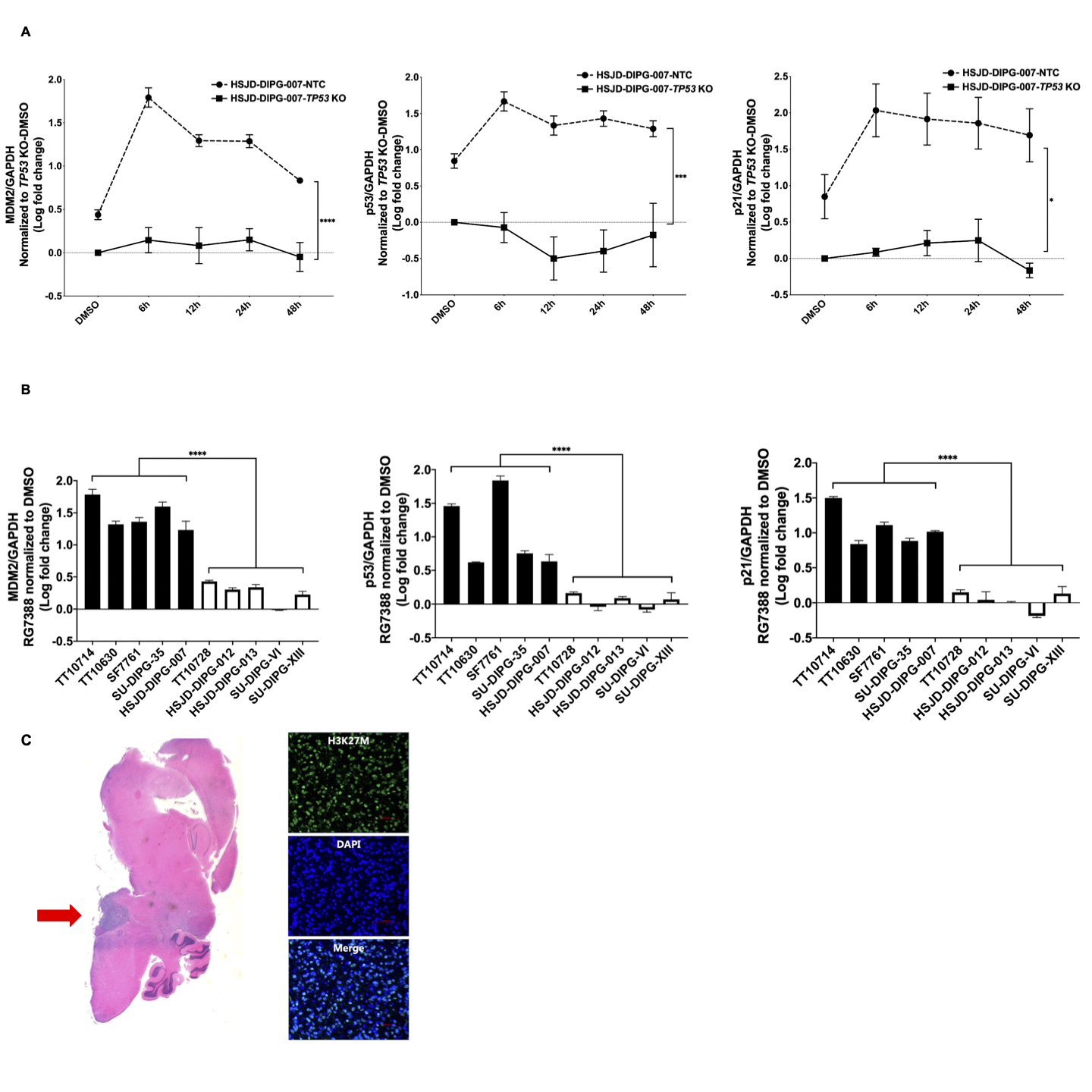

Supplement: Supplementary file 4 — Additional file 4: Figure S4. (A) Quantification of protein expression level of MDM2, p53 and p21 in HSJD-DIPG-007-NTC and HSJD-DIPG-007-TP53 KO treated with DMSO or 25nM RG7388 for 6 hours, 12 hours, 24 hours and 48 hours. Mean ± SEM, n=3 independent studies for each condition. P-values based on Two-way ANOVA. *P < 0.05. ***P < 0.001. ****P < 0.0001. (B) Quantification of protein expression level of MDM2, p53 and p21 in five TP53 wild-type and five TP53 mutant DIPG cell lines treated with DMSO and 100nM RG7388 for 24 hours. Mean ± SEM, n=3 independent studies for each condition. P-values based on Student’s t-test. ****P < 0.0001. (C) Representative H&E and immunofluorescence staining of brainstem xenograft. Scale bar: 100μm. [file 40478_2021_1270_MOESM4_ESM.tiff]
