# Supplementary material for: TP53 wild-type/PPM1D mutant diffuse intrinsic pontine gliomas are sensitive to a MDM2 antagonist
Source: Acta Neuropathol Commun. 2021 Nov 3;9:178. doi: 10.1186/s40478-021-01270-y (PMC8565061; doi:10.1186/s40478-021-01270-y)
Supplement: Supplementary file 5 — Additional file 5: Supplementary Methods [file 40478_2021_1270_MOESM5_ESM.docx]

**Supplementary Methods**

**Western blot**

Cell pellets were lysed with Blue Loading Buffer Pack (Cell Signaling Cat#7722) and were sonicated using the Bioruptor UCD-200TM sonicator. Total cell lysate containing ~20 ug total protein was resolved using (4-12%) NuPAGE Bis-Tris gradient gel. Gels were soaked in NuPAGE protein transfer buffer and transferred to a PVDF membrane using a XCell II™ Blot Module. After transfer, PVDF membranes were washed briefly in TBST (150 mM NaCl, 50 mM Tris-HCl, pH7.5, 0.1% Tween-20) and then blocked for 1-2 hours in Pierce TBST Protein-Free blocking buffer (Cat#37571). After blocking, primary antibodies were diluted in TBST blocking buffer and incubated overnight at 4°C. Membranes were washed and then incubated with horseradish peroxidase (HRP) conjugated secondary antibody for one hour and HRP signals were detected by chemiluminescence using the BioRad ChemiDoc MP system.

**RNA-seq data analysis**

Reads were trimmed using cutadapt (https://cutadapt.readthedocs.io/en/stable/) and mapped to hg38 genome using HISAT2^1^. FeatureCounts was used to quantify counts over reference genes^2^. Transcript level (FPKM) and DESeq2-normalized counts were performed using DESeq2^3^. DESeq2-normalized counts were then log2-transformed and plotted as Heatmaps using ggplot2-heatmap2^4^. EGSEA with 10 methods (camera, safe, gage, zscore, gsva, globaltest, ora, ssgsea, padog, plage, fry and roast) was used for enrichment analysis of KEGG pathways^5^. Differential expression (from DESeq2) was plotted as volcano plots using ggplot2-volcanoplot package^4^.

***In vivo* efficacy on mouse models**

12 outbred athymic nude mice (J:NU Stock#007850) were treated with RG7388 delivered by oral gavage at 50 mg/kg. Two mice were randomly selected and sacrificed at six time points 0.5 hour, 1 hour, 2 hours, 4 hours, 8 hours and 24 hours after the treatment. We collected plasma, cerebrospinal fluid (CSF), cerebral hemispheres tissues and brainstem tissues from the mice. Collected tissues were mixed with water (4X weight) and homogenized using H-speed disperser (ULTRA-TURRAX, Model T10). Proteins in Plasma, CSF and tissue homogenates were precipitated by MeOH/Acetonitrile (1:1, v/v). Supernatants were collected and analyzed by Mass Spectrometry (AB Sciex 5500). The concentration of RG7388 was quantified by Analyst Software 1.6.3.

To further access the *in vivo* efficacy of RG7388 on orthotopic DIPG models, HSJD-DIPG-007 cells were transduced with CMV-Firefly luciferase lentivirus (Cellomics Technology cat#C839R47). Stereotactic injection equipment was used, and the coordinate was 1.5mm posterior to Lambda, 1.0mm lateral to midline and 4.5mm beneath the skull. 5 x 10^5^ cells were injected into the brainstem of six to eight weeks old nude mice (n=18) from The Jackson Laboratory. Every three days, mice were injected with D-Luciferin (GoldBio cat#LUCNA) and luminescence signals were monitored using IVIS Spectrum In Vivo Imaging System. On the 21st-day post injection, mice with similar bioluminescence signals were randomly assigned to vehicle group or RG7388 treatment group (n=18). For each group, mice were treated by oral gavage with 50mg/kg of RG7388 (AstaTech cat#40916) dissolved in (2-Hydroxypropyl)-β-cyclodextrin solution (MilliporeSigma cat#332607) or vehicle once per day, five days per week for three weeks. Mice were assessed for neurological symptoms and weight loss and sacrificed when either 20% weight loss was measured or when they exhibited neurologic symptoms. Kaplan–Meier curves were used for survival analysis.

**H&E and immunofluorescence staining**

Standard H&E staining procedures were performed, requiring paraffin sections to undergo deparaffinization in 3 washes with Sub-X (Leica Cat#3803670) followed by rehydration in a graded ethanol series, Hematoxylin staining, wash with ammonia water, eosin staining, dehydration in a graded ethanol series, and clearing in Sub-X followed by mounting with a Sub-X-based mounting media (Leica Cat#3801740). Standard immunofluorescent staining procedures were performed^6^. Primary antibodies: H3K27M (Cell Signaling Cat#74829, 1:1000) and p21 (Cell Signaling Cat#2947, 1:1000). Secondary antibody: anti-rabbit IgG Alexa Fluor 488 (Thermo Fisher Scientific Cat#A27034, 1:2000). Cells were stained with DAPI (Millipore Sigma Cat#D9542, 0.5μg/mL). Immunofluorescent images were imaged on the Zeiss 880 at Duke Light Microscopy Core Facility. Positively stained cells were both manually counted and counted with ImageJ. Those assigned to counting were blinded to animal genotype and were given designated quadrants of specific size and magnification to count.

**Reference**

**1.** Kim D, Landmead B, Salzberg SL. HISAT: a fast spliced aligner with low memory requirements. *Nat Methods.* 2015; 12(4):357-U121.

**2.** Liao Y, Smyth GK, Shi W. featureCounts: an efficient general purpose program for assigning sequence reads to genomic features. *Bioinformatics.* 2014; 30(7):923-930.

**3.** Love MI, Huber W, Anders S. Moderated estimation of fold change and dispersion for RNA-seq data with DESeq2. *Genome Biol.* 2014; 15(12).

**4.** Wickham H. ggplot2: Elegant Graphics for Data Analysis. *Use R.* 2009:1-212.

**5.** Alhamdoosh M, Ng M, Wilson NJ, Sheridan JM, Huynh H, Wilson MJ, et al. Combining multiple tools outperforms individual methods in gene set enrichment analyses. *Bioinformatics.* 2017; 33(3):414-424.

**6.** Wang CC, Bajikar SS, Jamal L, Atkins KA, Janes KA. A time-and matrix-dependent TGFBR3-JUND-KRT5 regulatory circuit in single breast epithelial cells and basal-like premalignancies. *Nat Cell Biol.* 2014; 16(4):345-+.
