# Supplementary material for: TP53 wild-type/PPM1D mutant diffuse intrinsic pontine gliomas are sensitive to a MDM2 antagonist
Source: Acta Neuropathol Commun. 2021 Nov 3;9:178. doi: 10.1186/s40478-021-01270-y (PMC8565061; doi:10.1186/s40478-021-01270-y)
Supplement: Supplementary file 6 — Additional file 6: Supplymentary Table S1. CLA analysis of tem DIPG cell lines [file 40478_2021_1270_MOESM6_ESM.docx]

**Supplementary Table S1: CLA analysis of ten DIPG cell lines**

|  | **AMEL** | **CSF1PO1** | **D13S317** | **D16S539** | **D21S11** | **D5S818** | **D7S820** | **TH01** | **TPOX** | **vWA** |
| --- | --- | --- | --- | --- | --- | --- | --- | --- | --- | --- |
| **TT10714** | X,X | 8,13 | 8,11 | 11,12 | 28,30 | 11,13 | 8,11 | 9,9 | 8,9 | 16,17 |
| **TT10728** | X,X | 11,12 | 8,11 | 12,12 | 30,30 | 10,11 | 8,11 | 9,9 | 11,11 | 17,18 |
| **TT10630** | X,X | 11,13 | 8,8 | 10,12 | 31.2,33,2 | 9,11 | 8,13 | 7,8 | 11,11 | 16,18 |
| **SF7761** | X,X | 12,12 | 8,9 | 11,11 | 28,31.2 | 11,12 | 10,11 | 7,9.3 | 11,12 | 14,18 |
| **HSJD-DIPG-007** | X,Y | 10,10 | 13,13 | 13,14 | 29,30 | 13,13 | 9,12 | 6,9.3 | 8,8 | 16,17 |
| **HSJD-DIPG-012** | X,Y | 12,12 | 10,11 | 11,12 | 28,30 | 11,12 | 10,11 | 8,8 | 9,11 | 16,18 |
| **HSJD-DIPG-013** | X,X | 11,12 | 8,11 | 12,12 | 29,34,2 | 11,12 | 10,12 | 7,7 | 8,11 | 13,18 |
| **SU-DIPG-VI** | X,X | 10,11 | 11,11 | 13,13 | 29,30,31 | 10,12 | 8,9 | 7,8 | 8,11 | 18,18 |
| **SU-DIPG-XIII** | X,X | 9,10 | 11,11 | 11,12 | 30,31 | 12,12 | 9,9 | 6,6 | 8,9 | 18,19 |
| **SU-DIPG 35** | X,X | 10,12 | 12,13 | 9,12 | 31.2,32.2 | 12,12 | 10,13 | 6,7 | 8,8 | 15,16 |
| **HSJD-DIPG-007-NTC** | X,Y | 10,10 | 13,13 | 13,14 | 29,30 | 13,13 | 9,12 | 6,9.3 | 8,8 | 16,17 |
| **HSJD-DIPG-007-P53KO** | X,Y | 10,10 | 13,13 | 13,14 | 29,30 | 13,13 | 9,12 | 6,9.3 | 8,8 | 16,17 |
