# Supplementary material for: TP53 wild-type/PPM1D mutant diffuse intrinsic pontine gliomas are sensitive to a MDM2 antagonist
Source: Acta Neuropathol Commun. 2021 Nov 3;9:178. doi: 10.1186/s40478-021-01270-y (PMC8565061; doi:10.1186/s40478-021-01270-y)
Supplement: Supplementary file 7 — Additional file 7: Supplymentary Table S2. Primers for Sanger sequencing [file 40478_2021_1270_MOESM7_ESM.docx]

**Supplementary Table S2: Primers for Sanger sequencing**

|  | **Forward** | **Reverse** |
| --- | --- | --- |
| **H3F3A** | GGTAAAGCACCCAGGAAGCA | ACATACAAGAGAGACTTTGTCCC |
| **TP53-Exon 1** | TTCCACCCCAAAATGTTAGTATCTA | TCCCAACAATGCAACTCCTATGATG |
| **TP53-Exon 2-3** | CACTGGCATGGTGTTGGGGGAG | TGTAGATGGGTGAAAAGAGCAGTCA |
| **TP53-Exon 4** | GGACTGACTTTCTGCTCTTGTCTTT | CAGAGATCACACATTAAGTGGGTAA |
| **TP53-Exon 5** | CTCTCTAGCTCGCTAGTGGGT | CGAAAAGTGTTTCTGTCATCCAAAT |
| **TP53-Exon 6** | GCCATGGCCATCTACAAGCA | TGGGGTTATAGGGAGGTCAAA |
| **TP53-Exon 7** | ACAGGTCTCCCCAAGG | AAACTGAGTGGGAGCAGTAAGGAGA |
| **TP53-Exon 8-9** | GGACAAGGGTGGTTGGGAGTAGA | CCCAATTGCAGGTAAAACAGTCAAG |
| **TP53-Exon 10** | CAGTTTCTACTAAATGCATGTTGCT | ATACACTGAGGCAAGAATGTGGTTA |
| **TP53-Exon 11** | CATCTTGATTTGAATTCCCGTTGT | CACCAGTGCAGGCCAACTTGTTCAG |
| **PPM1D-Exon 6** | ACCCCGTTTTTGCCATCCTA | AACCTCATTTCCCAGATGCA |
